# Central toxic effects of an antitumoral bufadienolide: In silico targets and in vivo findings

**DOI:** 10.1007/s00210-026-05462-y

**Published:** 2026-05-26

**Authors:** Rayran Walter Ramos de Sousa, Antonia Amanda Cardoso de Almeida, Gabriel da Silva Fernandes, Rita de Cassia de Lima Sousa, Railson Pereira Souza, Layana Karine Farias Lima, Francisco das Chagas Pereira de Andrade, Anderson Nogueira Mendes, Evaldo dos Santos Monção Filho, Mariana Helena Chaves, Gerardo Magela Vieira Junior, Domingos de Jesus Rodrigues, Dalton Dittz, Chistiane Mendes Feitosa, Paulo Michel Pinheiro Ferreira

**Affiliations:** 1 Laboratory of Experimental Cancerology (LabCancer), Department of Biophysics and Physiology, Federal University of Piauí, Universitária Avenue, Ininga, Teresina, Piauí 64049-550 Brazil; 2 Department of Chemistry and Biology, State University of Maranhão, Caxias, Brazil; 3 Laboratory of Natural Products and Experimental Neurochemistry (Lpnnex), Department of Chemistry, Federal University of Piauí, Teresina, Brazil; 4 Laboratory of Innovation in Science and Technology (Lacitec), Department of Biophysics and Physiology, Federal University of Piauí, Teresina, Brazil; 5 Laboratory of Natural Products (Lpn), Department of Chemistry, Federal University of Piauí, Teresina, Brazil; 6 Institute of Natural, Humanities and Social Sciences, Federal University of Mato Grosso, Sinop, Brazil; 7 Laboratory of Antineoplastic Pharmacology (Lafan), Department of Biochemistry and Pharmacology, Federal University of Piauí, Teresina, Brazil

**Keywords:** Cardiotonic steroid, Glutamate receptor, Neurotoxicity, Marinobufagin, Seizures, Sodium channels

## Abstract

Marinobufagin (MBG), a cardiotonic steroid from *Rhinella* toads, exhibits potent antitumor activity. However, their involvement in the mammalian neurotoxicity remains unclear. Therefore, this study evaluated the systemic (neuro)toxicological action of MBG to understand its central effects, particularly those associated with seizure-inducing activity. Initially, physicochemical, pharmacokinetic, and drug-likeness profiles and molecular docking with receptors/channels were investigated by in silico platforms. Next, the pro-convulsive action of MBG was investigated using pharmacological modulators for specific neurotransmission pathways and in vitro inhibitory activity of acetylcholinesterase was quantified. MBG has intestinal absorption and capacity to cross the blood–brain barrier (BBB), binds strongly to plasma proteins, and acts as an inhibitory substrate for the CYP3A4 enzyme. It was classified as a drug-like molecule according to Lipinski’s rule and ineligible according to the Lead-like and World Drug Index criteria. Molecular docking emphasized the interaction of MBG with ictogenesis-related targets, confirmed by reduction of in vivo seizures and death of pretreated animals with pharmacological blockers for dopamine D2 and glutamate NMDA receptors and Na_v_1.2 channels, mainly. It was shown that affinity of MBG for excitatory targets is essential for neuronal excitability and onset of seizures and that interaction with GABA_A_ receptors is involved in bufadienolide-induced lethality. The findings also suggest that MBG-induced seizures may involve in silico binding to NMDA receptors and interactions with key excitatory (Na_v_1.2 channels and NMDA receptor) and inhibitory (GABA_A_ and D2 receptors) neuronal targets, contributing to altered neuronal excitability. Notably, it is the first report that characterized MBG-induced seizures and proposes it as a promising chemical option to understand ictogenesis and mechanism(s) of new anticonvulsive agents.

**Supplementary Information:**

The online version contains supplementary material available at 10.1007/s00210-026-05462-y.

## Introduction

Bufadienolides are plants’ or animals’ molecules chemically characterized by the presence of a steroidal and a lactone ring. They are commonly found in Bufonidae cutaneous secretions stored in parotoid glands as a biochemical defense against predators (Deng et al. 2020; Iguchi et al. 2020; El-Seedi et al. 2022; Rodriguez et al. 2023). The genus *Rhinella*, for example, is notable for species and molecular diversity and has broad geographic distribution within the American continents (Sousa et al. 2017; Pereyra et al. 2021; Kowalski et al. 2023).

Among the molecules, it highlights resibufagin, telocinobufagin, bufalin, cinobufagin, arenobufagin, and marinobufagin (MBG) (Ferreira et al. 2013; Botelho et al. 2019; Sinhorin et al. 2020; Zhan et al. 2020; Zou et al. 2022). MBG is found in skin secretions of toad species (e.g., *Rhinella rubescens*, *R. marina*, *R. schneideri*, *Rhaebo guttatus*, *R. margaritifera*, *R. jimi*, and *R. granulosa*) (Cunha-Filho et al. 2005, 2010; Abdelfatah et al. 2019; Garcia et al. 2019; Oliveira et al. 2019; Sinhorin et al. 2020; Monção Filho et al. 2021a; Barros et al. 2023) and, like other bufadienolides, it is recognized for cardiotonic activity because of its ability for inhibiting Na^+^/K^+^-ATPase pump (Sousa et al. 2017; Amaral et al. 2018; Carullo et al. 2023). Additionally, studies have demonstrated that MBG exhibits potent cytotoxic activity against tumor lines (Ferreira et al. 2013, 2023; Machado et al. 2018; Monção Filho et al. 2021b), in vivo antitumor effects on glioma (Lan et al. 2018) and colorectal carcinoma (Ferreira et al. 2023), antimicrobial activity upon *Staphylococcus aureus* and *Escherichia coli* (Cunha-Filho et al. 2005), antiparasitic action against *Plasmodium falciparum* (Banfi et al. 2021), and anti-inflammatory effects (Carvalho et al. 2019).

Findings conducted by our research group revealed the occurrence of MBG-induced in vivo seizures after intraperitoneal injections (Ferreira et al. 2023). Seizures are characterized by electrical conductance irregularities in the central nervous system (CNS), resulting from atypical, transient, and synchronous electrophysiological events that increase the excitability of specific regions or induce generalized activation (Fisher et al. 2017; Rai et al. 2017; Beniczky et al. 2025). This phenomenon is primarily associated with neurological disorders such as chronic epilepsy but may also arise from distinct etiological factors including trauma, infections, tumors, and psychogenic stimuli. Clinical manifestations of seizures include involuntary muscle contractions, tremors, loss of consciousness, and memory impairment (Brown and Reuber 2016; Gasparini et al. 2019; Anwar et al. 2020; Vera-González 2022). Only epilepsy affects over 50 million people worldwide (Feigin et al. 2025). Moreover, it is estimated that more than half of people with this disease do not receive health services in Latin America and the Caribbean (PAHO 2019).

Currently, it is well established that the pathophysiology of seizures and epilepsy directly involves multiple neurotransmission systems, including GABAergic, glutamatergic, serotonergic, cholinergic, noradrenergic, dopaminergic, opioidergic, histaminergic, and endocannabinoid pathways, and relies on the active participation of ion channels (Na^+^, K^+^, and Ca^2+^) (Freitas 2011; Rowley et al. 2012; Kow et al. 2014; Brodie et al. 2016; Rosenberg et al. 2017; Tiwari et al. 2019; Akyuz et al. 2021; Sugaya and Kano 2022; Yang et al. 2022; Debanne et al. 2024). In view of such complexity, there is a growing search for novel therapeutic options, as well as to understand pathogenicity aspects (Bhuvanendran et al. 2018; Löscher and White 2023).

Besides, current literature still lacks comprehensive studies about in vivo toxicity of bufadienolides, particularly on the central nervous system (CNS) (Ferreira et al. 2023). Indeed, it is well established that certain bufadienolides can target multiple extracellular and intracellular sites, including voltage-gated calcium channels (VGCCs) (Bick et al. 2002), potassium channels (Xie et al. 2000), and the sodium–potassium pump (Wang et al. 2014; Sousa et al. 2017; Oliveira et al. 2018). However, their involvement in the mammalian neurotoxicity remains unclear. In this context, we hypothesize that MBG induces seizure behavior by modulating ictogenesis-related pharmacological targets, such as muscarinic, GABAergic, glutamatergic, opioid, dopaminergic, serotonergic receptors, and sodium and calcium ion channels. Therefore, the present study evaluated systemic (neuro)toxicological action of MBG to understand its central effects, particularly those associated with seizure-inducing activity.

## Materials and methods

### Collection, isolation, and identification of marinobufagin

The collection of *Rhinella marina* skin secretions was carried out in Sinop, Mato Grosso, Brazil. The animals were identified by Professor Domingos de Jesus Rodrigues and specimens of *R. marina* (voucher ABAM-H 1262) were deposited in the Biological Collection of Southern Amazonia (Sinop, Mato Grosso, Brazil). This research was registered at SisGen (National System for the Management of Genetic Heritage and Associated Traditional Knowledge: #AE58A09) in accordance with Brazilian legislation for accessing genetic resources and associated traditional knowledge (Law 13.123/2015). Extraction and identification of 3β,5β-dihydroxy-14,15-epoxy bufadienolide (MBG) was performed as previously described (Kerkhoff et al. 2016; Ferreira et al. 2023).

### Computational prediction of physicochemical and pharmacokinetic properties

**Fig. 1:**
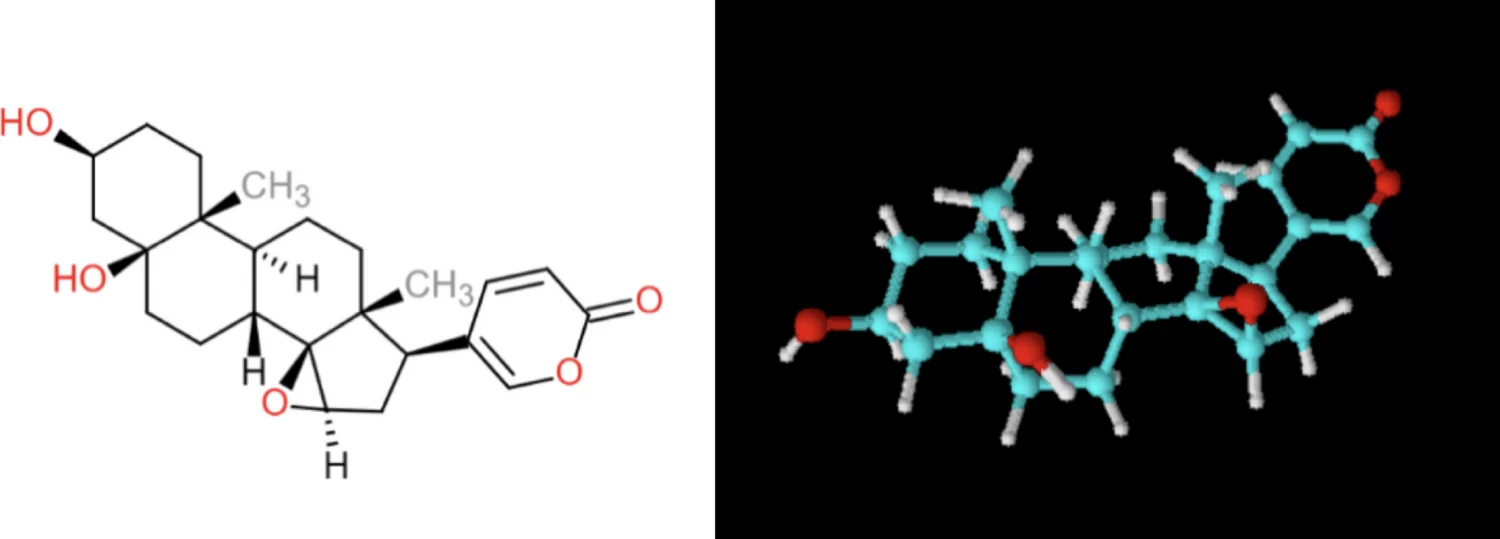
Molecular structure of marinobufagin in 2D and 3D views

The 2D and 3D structures (Fig. 1) and the *SMILES* notation of the molecule (C_24_H_32_O_5_) used for in silico analysis [absorption, distribution, and metabolism (ADME) and target prediction] were obtained and downloaded from the PubChem database (https://pubchem.ncbi.nlm.nih.gov).

To predict physicochemical properties, pharmacokinetic parameters, and drug-likeness profiles (Lipinski’s rule, CMC-like, MDDR-like, Lead-like, and WDI-like), the platforms PreADMET (https://preadmet.webservice.bmdrc.org/) and SwissADME (http://www.swissadme.ch/) were employed (Andrade and Mendes 2020; Nunes et al. 2020), with complementary data visualized through the Bioavailability Radar and the BOILED-Egg model. Moreover, SwissTargetPrediction (https://www.swisstargetprediction.ch/) was used to predict macromolecular targets of the compound (Souza et al. 2024).

### Induction of marinobufagin seizures and neuropharmacological mechanisms

#### In vivo investigation

In order to evaluate the pro-convulsive action of MBG, animals were randomly divided into groups (*n* = 7 animals/group). Negative control group received i.p. DMSO 5% in saline as vehicle and the experimental group was injected with an i.p. single dose of MBG 10 mg/kg. This dose was based on a previous study conducted by Ferreira et al. (2023), who firstly reported seizure episodes during hippocratic screening analyses.

The involvement of specific neurotransmission pathways and participation of ion channels was investigated using reference drugs (Table 1) and based on the neuropharmacological study of Freitas (2006). Each drug was i.p. injected 30 min prior to seizure induction with MBG. Following MBG injection, animals were followed up for 4 h and parameters such as latency for the first seizure, absolute and relative frequency of seizure occurrence, number of surviving animals, and mortality were recorded.

**Table 1:** Reference drugs used to investigate underlying mechanisms of marinobufagin-induced seizures

Neurotransmission pathway or ion channel	Drug	Single dose (mg/kg)
Cholinergic	Atropine	25
Dopaminergic	Haloperidol	10
Serotonergic	Fluoxetine	10
GABAergic	Diazepam	5
	Clonazepam	2
Glutamatergic	Ketamine	0.5
Opioidergic	Morphine	0.1
Na^+^ channel	Carbamazepine	20
Ca^2+^ channel	Gabapentin	150

#### In vitro activity of acetylcholinesterase

Using 96-well microplates, 25 µL of MBG (0.3125 to 10 µg/mL) were mixed with 25 µL of AChE (0.22 U/mL in Tris–HCl 50 mM with BSA 1%, pH 8) and 50 µL of buffer (50 mM Tris–HCl with BSA 1%, pH 8), followed by incubation for 5 min at 37 °C. Subsequently, 125 µL of 3 mM 5,5′-dithiobis-(2-nitrobenzoic acid) diluted in Tris–HCl (pH 8), 0.1 mol/L NaCl, 0.02 mol/L MgCl_2_, and 25 µL of acetylthiocholine iodide (15 mM in water) were added for the first absorbance reading. A second reading was performed after 5 min.

Negative control represented 100% of AChE activity and consisted of a mixture of 25 µL buffer (50 mM Tris–HCl, pH 8) with methanol 10%. Rivastigmine (50 µg/mL) was used as a positive control. Absorbance readings were performed using a microplate spectrophotometer (GloMax Discover Microplate Reader) at 405 nm. The experiment was conducted in triplicate and the percentage of AChE inhibition [*I* (%)] was calculated as follows (Dohi et al. 2009): *I* (%) = 1 − (*A*_f_ − *A*_i_)/(*A*_fCN_ − *A*_iCN_) × 100, where *A*_f_ and *A*_i_ represent the final and initial absorbance values of the samples, and *A*_fCN_ and *A*_iCN_ correspond to the final and initial absorbance values of the negative control.

### Computational docking studies

Docking simulations were carried out using AutoDock Vina (Trott and Olson 2009). Molecular targets and Protein Data Bank (PDB) codes were obtained by electron microscopy [voltage-gated sodium channel Na_v_1.2 (6J8E, resolution 3 Å)] or X-ray diffraction [dopamine D_2_ receptor (6CM4, 2.867 Å), GABA_A_ receptor (5OSA, 2.75 Å), and NMDA receptor GluN2A subunits (4NF15 and 6E7R, 1.903 Å and 2.1 Å, respectively)]. Protein structures were collected from the PDB with resolutions up to 3.0 Å and all crystallographic ligands and water molecules were removed. Polar hydrogens were added and protonation states were adjusted according to physiological conditions using AutoDock Tools (Forli et al. 2016; Araújo et al. 2017). Ligand structures were energy-minimized in Discovery Studio 2019 and PDBQT files were generated for both ligands and receptors. The docking grid box was centered on the binding site and defined to fully cover the active pocket. The exhaustiveness parameter was set to 100 to ensure reliable conformational sampling. Binding modes were analyzed and visualized in 2D and 3D interaction maps (PyMol and Discovery Studio 2019). Docking protocol was validated by “redocking” experiments and root mean square deviation (RMSD) values below 2.0 Å were considered indicative of reliable docking accuracy.

## Statistical analysis

For results obtained expressed as mean ± standard error of the mean (S.E.M.), Shapiro–Wilk tests were performed to evaluate data normality and to guide the choice between parametric and nonparametric analyses. Next, data were analyzed by one-way ANOVA followed by Tukey for multiple comparisons and seizure incidence was evaluated by the Fisher’s exact test. Differences were considered statistically significant when *p* < 0.05. All analyses were conducted in the free version of GraphPad Prism version 10.5.0 (GraphPad Software®).

## Results

### Toxicokinetic and pharmacokinetic parameters of marinobufagin

In silico analysis revealed physicochemical properties, pharmacokinetic profile, and drug-likeness prediction of MBG (Table 2). Regarding its physicochemical characteristics, MBG exhibits a molecular weight of 400.51 g/mol, with two hydrogen bond donors and five hydrogen bond acceptors, one rotatable bond, and a topological polar surface area (TPSA) of 82.20 Å^2^. Based on the Log S (intrinsic solubility) and Log P (partition coefficient: 1-octanol/water system) values of − 3.99 and 3.13, respectively, MBG is hardly insoluble in water.

**Table 2:** In silico properties of marinobufagin

Properties	Parameters	Prediction data
Physicochemical	Molecular weight (g/mol)	400.51
	Water solubility (Log S)	− 3.99
	Lipophilicity (Log P; XLOGP3)	3.13; 2.50
	Number of rotatable bonds	1
	Number of hydrogen bond donors	2
	Number of hydrogen bond acceptors	5
	Fraction of sp^3^ carbons	0.79
	Molar refractivity	108.86
	Topological polar surface area (Å^2^)	82.20
	Human intestinal absorption (%)	93.73
Pharmacokinetic	Caco-2 permeability coefficient (nm/s)	22.27
	MDCK permeability coefficient (nm/s)	4.29
	Plasma protein binding (%)	93.26
	Blood–brain barrier penetration	0.20
	P-glycoprotein inhibition	No
	CYP3A4 substrate	Yes
	CYP2C9 substrate	No
	CYP2D6 substrate	No
	CYP3A4 inhibition	Yes
	CYPC9 inhibition	No
	CYPD6 inhibition	No
Drug-likeness prediction	Lipinski’s rule of five	Qualified
	CMC-like rule	Qualified
	MDDR-like rule	Intermediate
	Lead-like rule	Disqualified
	WDI-like rule	Disqualified

Marinobufagin demonstrated high human intestinal absorption (93.7%) and apparent permeability (Papp) values for Caco-2 (human epithelial adenocarcinoma) and MDCK (Madin-Darby canine kidney) cells of 22.27 and 4.29 nm/s, respectively, indicating intermediate permeability (between 4 and 70 nm/s) (Table 2). It also exhibits strong plasma protein binding (binding rate of 93.26%) and a brain-to-plasma concentration ratio (C_brain_/C_plasma_) of 0.20 (Table 2). Based on this value, MBG has intermediate capacity to cross the blood–brain barrier (BBB), as the ratio falls within the range of 0.1 to 2.0. Meanwhile, it does not interact with P-glycoprotein (P-gp) but it acts as an inhibitory substrate for the CYP3A4 enzyme (Table 2).

Regarding drug-likeness prediction rules and corresponding databases, MBG was classified as a drug-like molecule according to Lipinski’s rule and the Comprehensive Medicinal Chemistry (CMC-like) database. It was considered intermediate under the Modern Drug Data Report (MDDR-like) rule and ineligible for to the Lead-like and World Drug Index (WDI-like) criteria (Table 2).

**Fig. 2:**
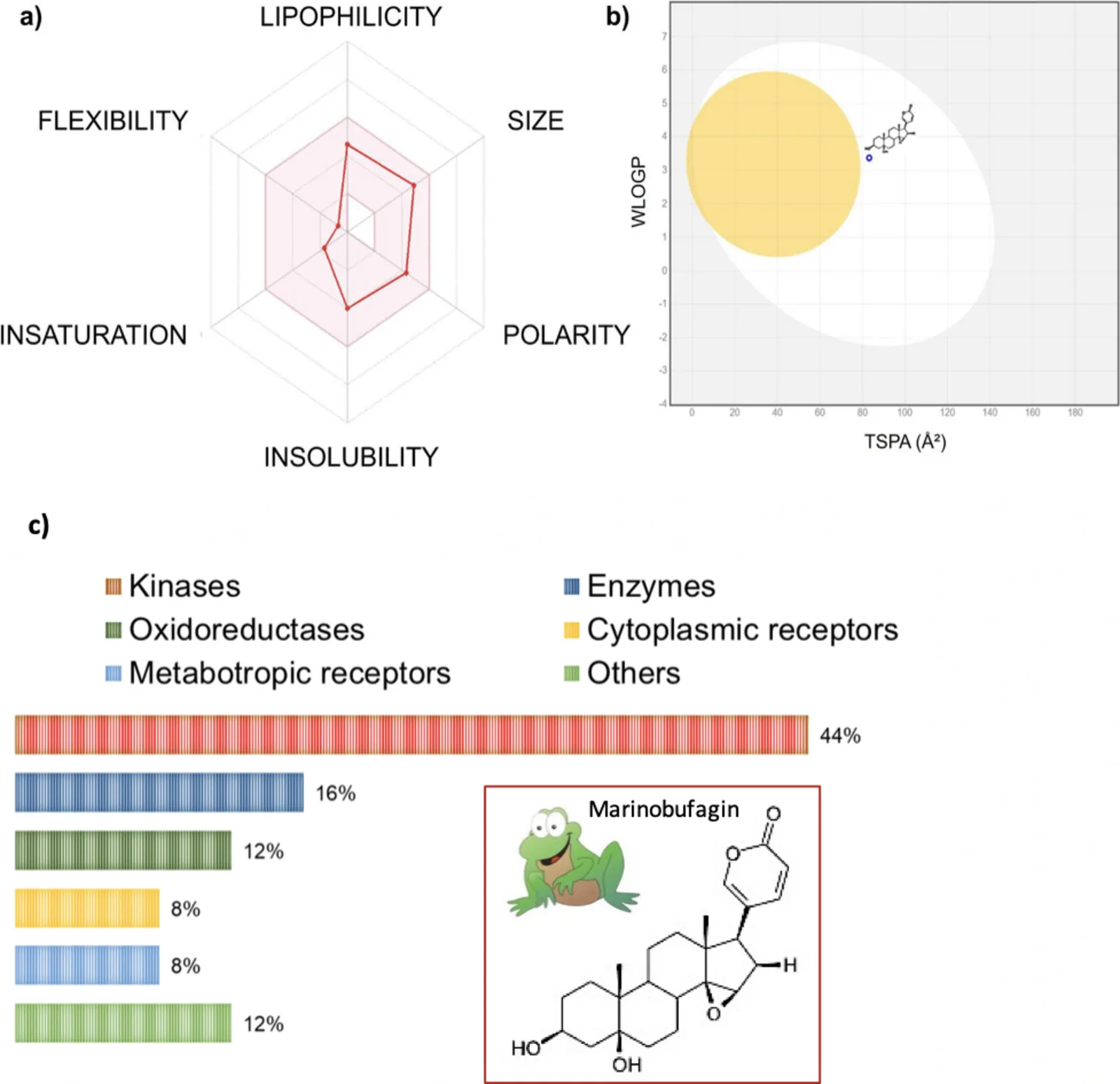
**a** Bioavailability radar of marinobufagin. The radar plot illustrates physicochemical properties of the compound, with the pink area representing the optimal range for oral bioavailability. **b** BOILED-Egg model for marinobufagin. **c** Computational prediction of probable marinobufagin’s pharmacotoxicological targets

The bioavailability radar (Fig. 2a) indicates MBG falls within the optimal range for a drug-like compound. This result is derived from key physicochemical parameters, including lipophilicity (XLOGP3 between − 0.7 and 5.0), solubility (Log S < 6), polarity, molecular weight (between 150 and 500 g/mol), molecular flexibility (rotatable bonds < 9), and saturation (fraction of sp^3^-hybridized carbons > 0.25). By meeting the established thresholds for each of these properties, MBG demonstrates favorable overall attributes for oral bioavailability.

BOILED-Egg analysis suggests that MBG exhibits good intestinal absorption but low probability for CNS penetration (Fig. 2b). Additionally, MBG was identified as a P-gp substrate, as indicated by the blue dot. It likely interacts with protein kinases (44%) predominantly, followed by general enzymes (16%), oxidoreductases (12%), cytoplasmic receptors (8%), metabotropic receptors (8%), and others (12%) (Fig. 2c).

### Marinobufagin-induced seizures can be prevented

All mice treated with a single dose of MBG (10 mg/kg, i.p.) exhibited seizure episodes, with a latency to the first convulsion of 240 ± 26.8 s (Table 3). All drugs used in the pretreatment demonstrated some degree of reduction of MBG-induced seizures. Specifically, morphine, fluoxetine, and clonazepam reduced seizures in 14%; atropine and haloperidol, 29%; gabapentin, 43%; and diazepam in 72%. Meanwhile, ketamine- and carbamazepine-pretreated animals did not show seizures (Table 3^3^).

**Table 3:** Effect of the pretreatment on marinobufagin-induced seizures

Group		Dose (mg/kg)	Seizures andincidence (%)	Latency for seizures (s)	Survival	Death (%)
Negative control		-	0/7 (0)	No seizures	7/7	0
Marinobufagin		10	7/7 (100)^#^	240 ± 26.8	3/7	57
Morphine	+ Marinobufagin	0.1	6/7 (86)	180 ± 26.8	6/7	57
Atropine		25	5/7 (71)	252 ± 22.5	7/7	14
Fluoxetine		10	6/7 (86)	430 ± 44.9	7/7	0
Haloperidol		10	5/7 (71)	430 ± 44.9^$^	7/7	0
Diazepam		5	2/7 (28)*	420 ± 60.0	7/7	0
Clonazepam		2	6/7 (86)	324 ± 88.2	7/7	0
Ketamine		0.5	0/7 (0)*	No seizures	7/7	0
Carbamazepine		20	0/7 (0)*	No seizures		0
Gabapentin		150	4/7 (57)	375 ± 78.9		0
Values are expressed as mean ± standard error of the mean (S.E.M.) (n = 7 animals/group). #p < 0.05 compared to the marinobufagin group and *p < 0.05 compared to the negative control group by the Fisher’s exact test. ^$^p < 0.05 compared with the marinobufagin group by one-way ANOVA followed by Tukey						

Four out of 7 animals died after a single dose of MBG 10 mg/kg (mortality rate of 57%). All drugs were able to completely prevent seizure-associated mortality when compared to the MBG alone, with exception for those animals pretreated with morphine (57%) and atropine (14%) (Table 3). However, only haloperidol-pretreated animals displayed increased latency to the first seizure (648 ± 61.2 s) in comparison with the MBG group (*p* < 0.05).

**Fig. 3:**
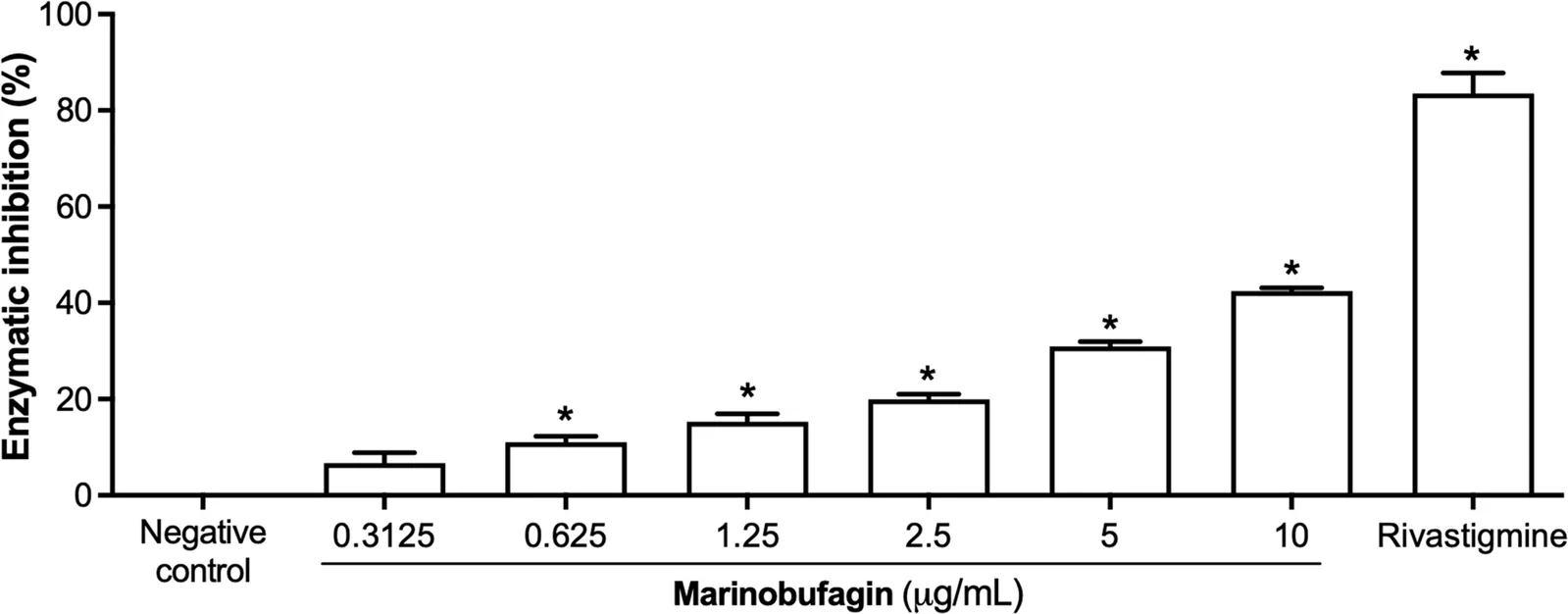
In vitro inhibitory activity of acetylcholinesterase enzyme promoted by marinobufagin isolated from *Rhinella marina* venom. Rivastigmine 50 µg/mL was used as a positive control. Columns are mean ± S.E.M. (*n* = 3). *p < 0.05 when compared to the negative control by one-way ANOVA followed by Tukey

In vitro assessments revealed that MBG significantly inhibited AChE in a concentration-dependent way (Fig. 3, *p* < 0.05). At the highest concentration tested (10 µg/mL), MBG inhibited 42.4% of enzymatic activity. Rivastigmine, used as a positive control at 50 µg/mL, inhibited 83.6% (*p* < 0.05).

### Marinobufagin has high affinity for nervous receptors

**Fig. 4:**
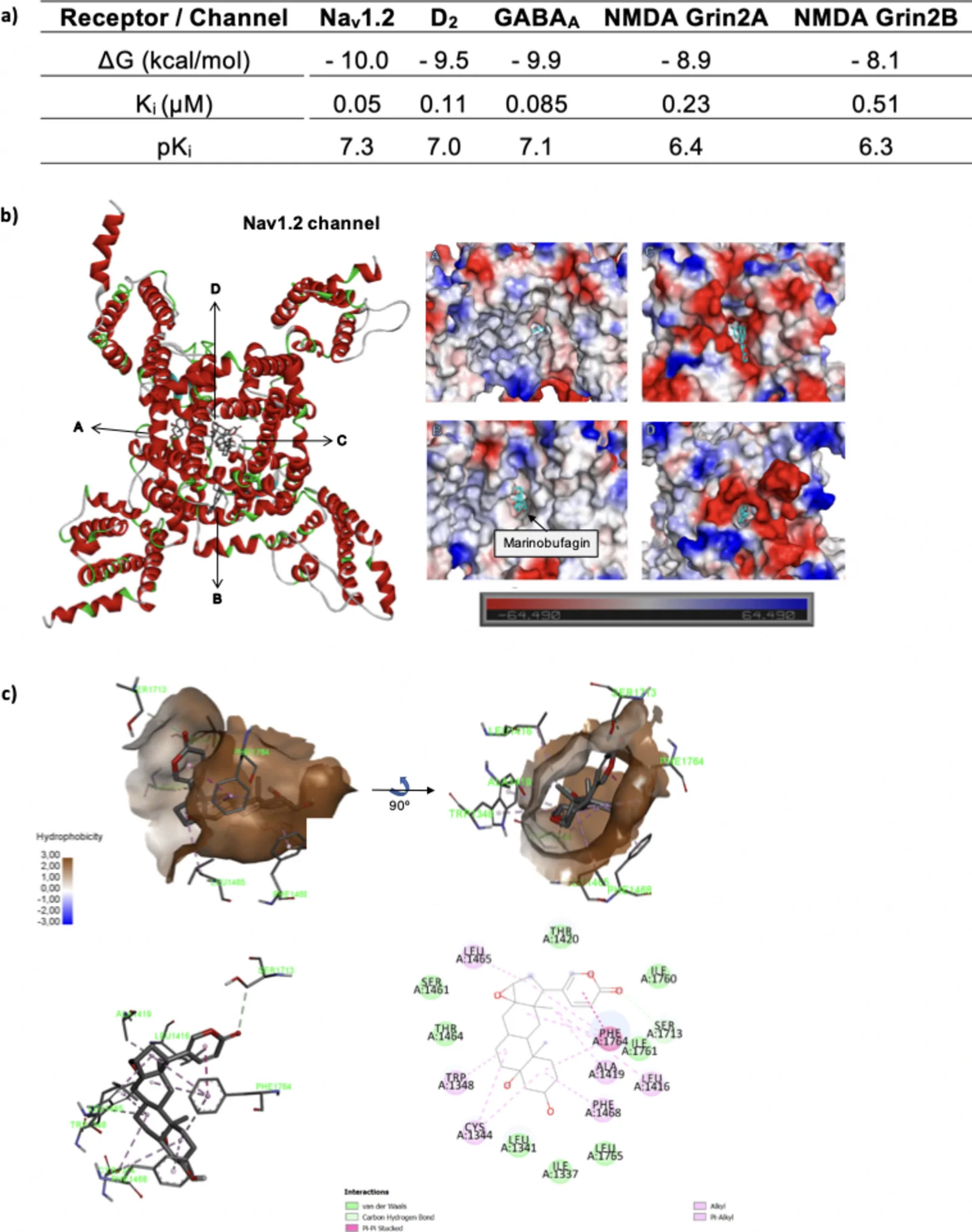
**a** Binding energy, inhibition constant, and pKi of marinobufagin complexes with receptors/channels; **b** 3D structure of Na_V_1.2 ribbons showing the sites filled by marinobufagin (left) and representation of electrostatic potential surfaces of the marinobufagin binding sites on the Na_V_1.2 protein calculated by the PyMol program (right): **c** surface area filled by marinobufagin in the selective cavity of Na_V_1.2 with frontal view of the site and 3D and 2D pharmacophore map showing the interactions of marinobufagin in the selective cavity of Na_v_1.2

Molecular docking results initially revealed that MBG can generate stable complexes with all investigated protein targets (Fig. 4). It was also found that the molecule has a higher affinity (Fig. 4a) for the Na_v_1.2 sodium channel (ΔG =  − 10.0 kcal/mol; Ki = 0.05 µM), followed by the GABA_A_ receptor (ΔG =  − 9.9 kcal/mol; Ki = 0.085 µM) and the dopaminergic D_2_ receptor (ΔG =  − 9.5 kcal/mol; Ki = 0.11 µM). For NMDA receptors, the affinity was slightly lower [ΔG of − 8.9 kcal/mol (Grin2A) and − 8.1 kcal/mol (Grin2B)] and Ki of 0.23 µM and 0.51 µM, respectively. Anyway, regarding pKi values, all exhibited values greater than 6.0, suggesting a biologically relevant affinity, especially for Na_v_1.2, GABA_A_, and D2, with pKi ≥ 7.0. RMSD is described in supplementary material.

Analyzing the molecular interactions of Na_v_1.2 channels (Fig. 4b and c), it was observed that MBG can bind to several functional sites. In the selective cavities (sites A and B), the interaction is mainly mediated by van der Waals with hydrophobic residues (Ile 1337, Leu 1765) and π-π T-shaped interactions (Phe 1764), and hydrogen-carbon (Ser 1713) and π-sigma (Val 406) bonds. In the intracellular (site C) and extracellular (site D) channels, conventional hydrogen bonds (Phe 1421, Gln 391) and electrostatic interactions, such as π-anion interactions with Lys 1422 residue, were noted. A coexistent occupation of these strategic sites may reflect a putative interaction with distinct regions of the channel.

**Fig. 5:**
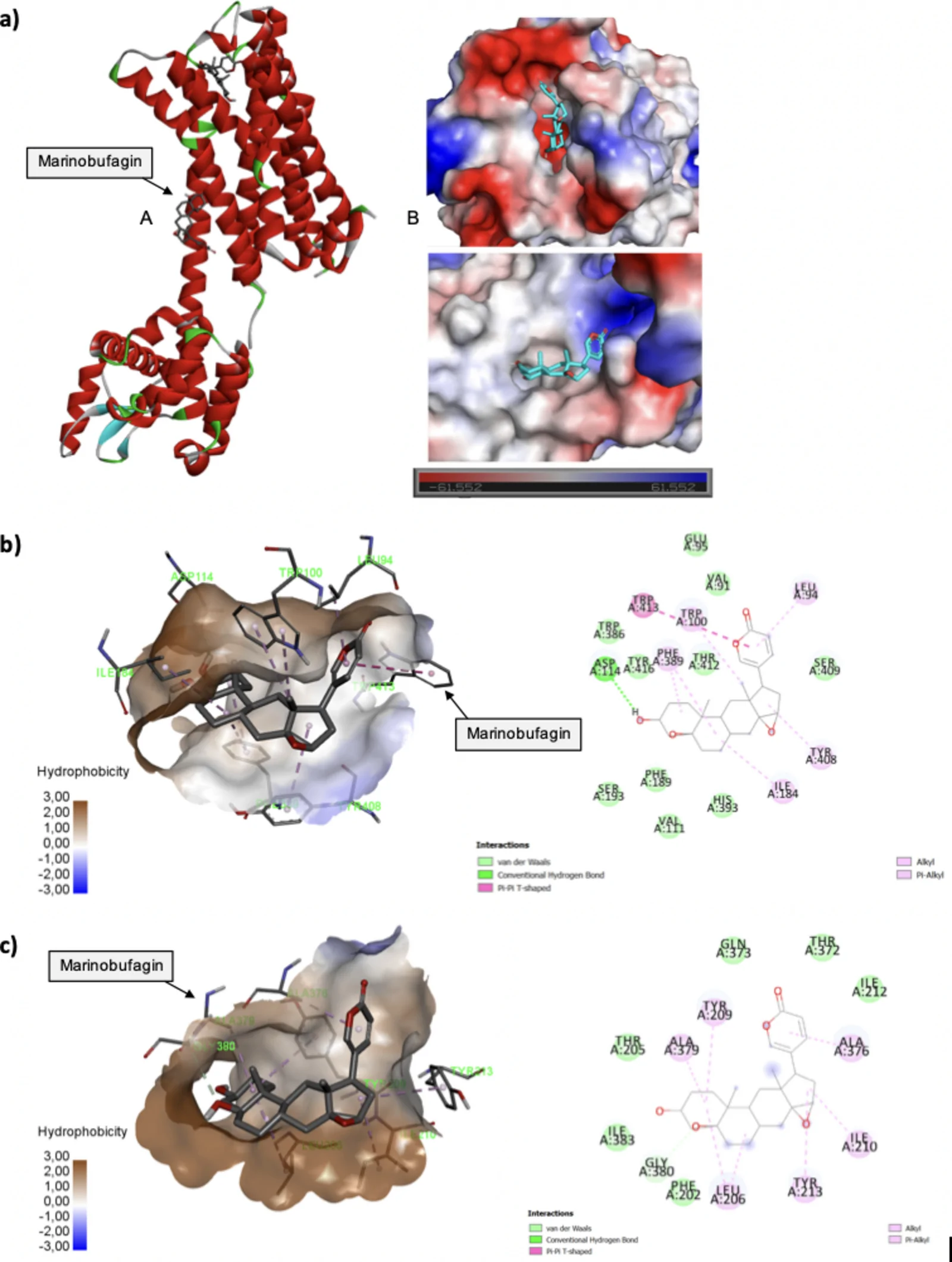
**a** 3D structure of the D_2_ dopamine receptor ribbons showing the sites filled by marinobufagin (A) and representation of electrostatic potential surface of the protein showing the interactions of marinobufagin in the active site channel (B); **b** 2D pharmacophore map showing the interactions of marinobufagin with the main hydrophobic active site cavity of the D_2_ dopamine receptor;** c** 2D pharmacophore map showing the interactions of marinobufagin with the lateral hydrophobic cavity of the D_2_ dopamine receptor

Regarding the interaction with the D2 dopaminergic receptor (Fig. 5), it was observed that MBG binds to both the main active site and a hydrophobic side channel. In the main active site, the compound creates a crucial hydrogen bond with Asp 114 and interacts with a “hydrophobic patch” built by the residues Trp 100, Ile 184, and Leu 94, stabilizing the ligand. Additionally, a π-π T-shaped interaction with Trp 413 reinforces the stability of the complex. In the side channel, the interactions are hydrophobic, predominantly, involving residues such as Ile 212 and Phe 202, and a hydrogen-carbon bond with Gly 380. Such dual binding may be associated with a distinct pharmacological profile, with consequences for selectivity and receptor binding kinetics.

**Fig. 6:**
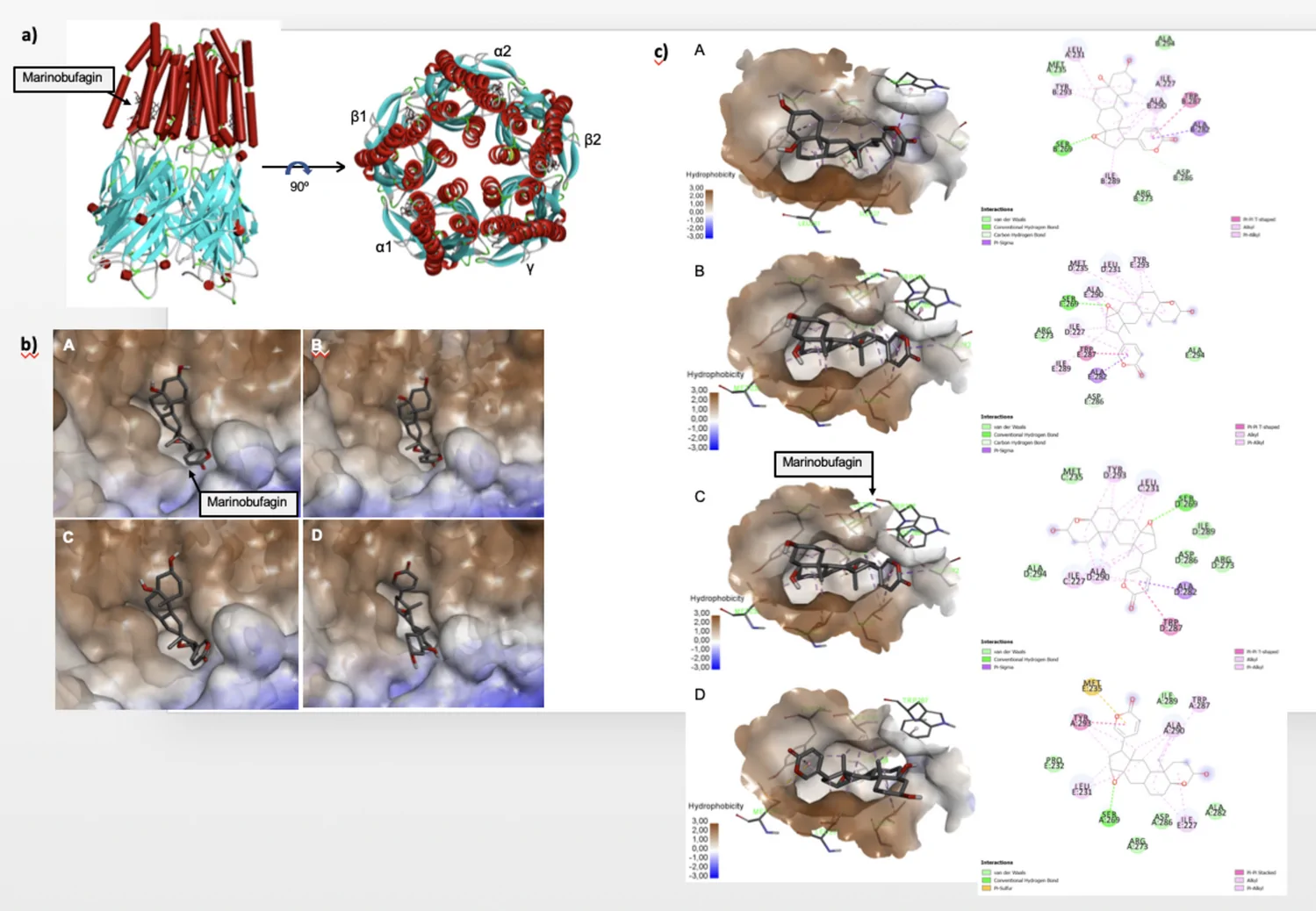
**a** 3D structure of GABA_A_ receptor rods showing the sites filled by marinobufagin (A); frontal view showing marinobufagin within the “grooves” formed at the junctions of the GABAA receptor side chains (B); **b** frontal view of the aqueous tunnel showing the marinobufagin binding site and the main sites involved in anesthetic effects; **c** surface of the cavity filled by marinobufagin in the GABA_A_ receptor (on the right) and 2D pharmacophore map showing the interactions of marinobufagin with the GABA_A_ receptor at sites A, B, C, and D (on the left)

MBG was also able to bind to four distinct sites (A, B, C, and D) of GABA_A_ subunits (Fig. 6). Hydrogen bonds with the residue Ser 269, a crucial site for allosteric modulation by neurosteroids and anesthetics, were evident at all sites. Furthermore, significant hydrophobic interactions were observed with Met 235 and Ala 290. The diversity of interactions, including π-sulfur (Met 235 at site D) and π-π T-shaped (Trp 287) bonds (Fig. 6c), indicates an allosteric modulation profile in a similar way found with benzodiazepines, acting at complementary sites though.

**Fig. 7:**
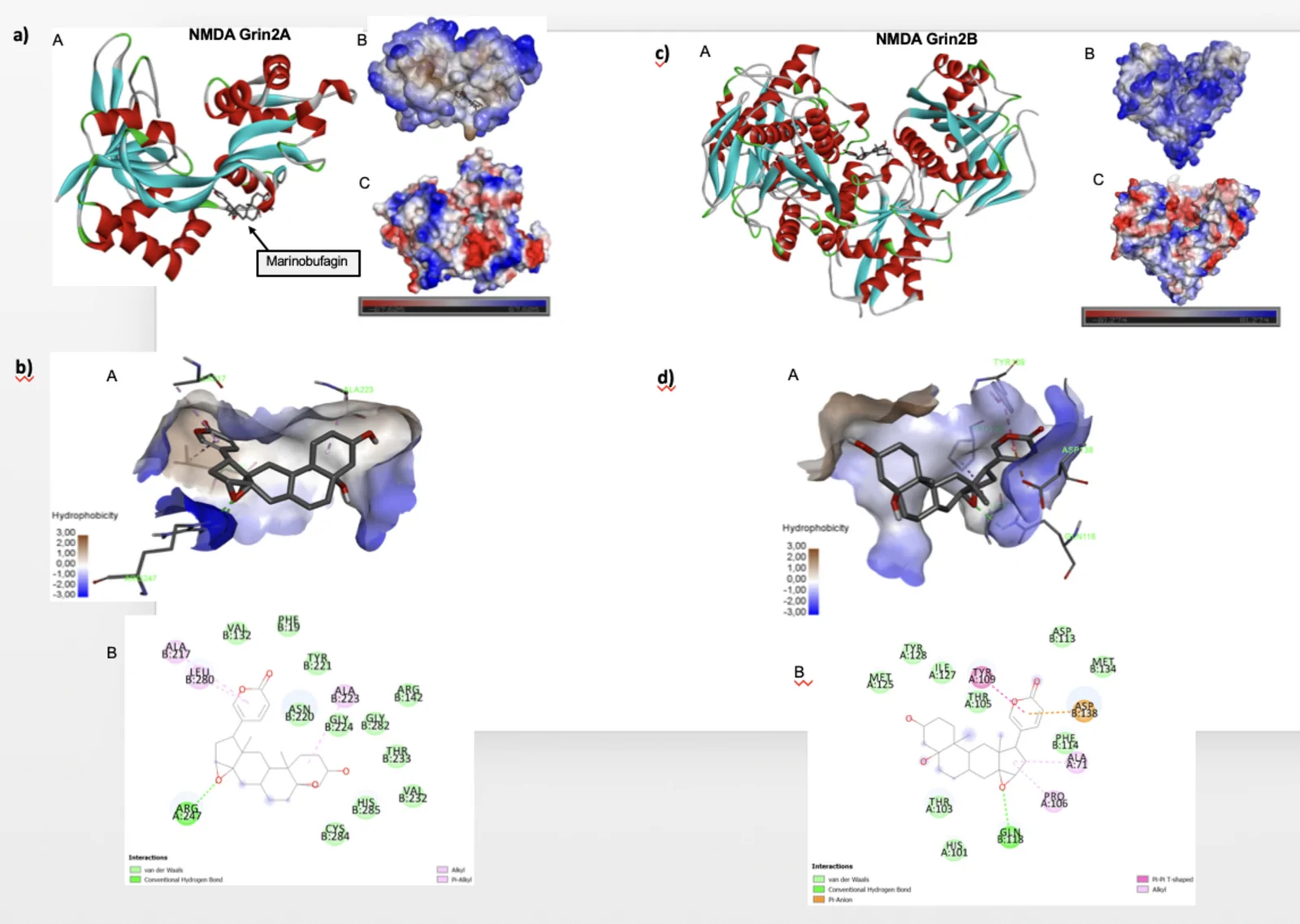
**a** and **c** 3D structure of NMDA Grin2A and Grin2B strands showing the sites filled by marinobufagin (A); hydrophobic surfaces of the B chain showing the location of marinobufagin in the protein (B); representation of the electrostatic potential surface of the protein showing marinobufagin sites in the active site channel (C); **b** and **d** side views of the surface of the extracellular cavity entrance occupied by marinobufagin in the NMDA Grin2A and Grin2B receptors (A); 2D pharmacophore map showing the interactions of marinobufagin with the NMDA Grin2A and Grin2B receptors obtained by virtual screening for better binding energies (B)

In the NMDA receptor Grin2A, MBG interacts (Fig. 7) (i) with the hydrophobic channel with the amino acids Arg 142, Asn 220, Cys 284, Gly 224, Gly 282, His 285, Phe 19, Val 132, Val 232, and Tyr 221 through van der Waals forces; (ii) with the fragments Ala 217, Ala 223, and Leu 280 through alkyl bonding; and (iii) with Arg 247 through hydrogen bond, with an ion energy of − 8.9 kcal/mol, demonstrating the ability to generate ligand–protein complexes (Fig. 4a). Meanwhile, MBG interaction with the amino-terminal domain of the GluN1–GluN2B NMDA receptor indicates a putative role for receptor modulation, showing interaction with (i) the hydrophobic channel of the critical binding site by the amino acid fragments Asp 113, Ile 127, Hist 101, Met 125, Met 134, Phe 114, Thr 103, Thr 105, and Tyr 128 via van der Waals forces; (ii) the fragments Ala 71 and Pro 106 via alkyl bond; and (iii) Gln 118 via hydrogen bond, and with Tyr 109 via π-π T-shaped linking (binding energy of − 8.1 kcal/mol).

A consistent translational pattern was observed with molecular targets. They exhibited stronger predicted binding affinities for MBG (ΔG ≤  − 8.0 kcal/mol) corresponding to those whose pharmacological modulation exhibited by anticonvulsive drugs resulted in prolonged latency to the first seizure and, in some cases, complete seizure blockade (Table 4). Notably, modulation of these same targets was also associated with 100% survival (Table 4).

**Table 4:** Translational comparison of marinobufagin predicted binding affinity to molecular targets and in vivo efficacy of anticonvulsant drugs in marinobufagin-induced seizures

Molecular targets for marinobufagin	ΔG (kcal/mol)	Latency increase (%)	Survival (%)
		Drug pretreatment + marinobufagin	
Na_v_1.2	− 10.0	No seizures	100
GABA_A_	− 9.9	90.5	100
D_2_ receptor	− 9.5	162.7	100
NMDA Grin2A	− 8.9	No seizures	100
NMDA Grin2A	− 8.1	No seizures	100

## Discussion

The physicochemical properties of a molecule and its corresponding biological performance enable the interpretation of pharmacodynamics, pharmacokinetics, and toxicity parameters. Therefore, the use of computational techniques to predict ADMET and physicochemical data and targets contributes to forecasting therapeutic efficacy and behavior of a prototype drug (Wenlock and Barton 2013; Ancuceanu et al. 2025). Here, we assessed the neurotoxic action of the bufadienolide MBG, taking into consideration computational and preclinical tools.

The lipophilicity of a bioactive compound is estimated by its Log P value, a critical parameter in medicinal chemistry for drug development, because it influences absorption, bioavailability, plasma protein binding, distribution, metabolism, and excretion processes, as observed in methyl succinimide derivatives (Kujawski et al. 2011; Kovačević et al. 2020). MBG exhibited good lipophilicity and low aqueous solubility, suggesting efficient passive diffusion following oral administration (Lipinski et al. 1997), which may confer favorable transmembrane permeability. Weng et al. (2011) and Yusuf et al. (2017) also described bufadienolides as poorly water-soluble and highly lipophilic compounds.

Such capacity to cross cell membrane supposes MBG has high potential for intestinal absorption in humans (Zhao et al. 2001; Khan et al. 2018). Previously, Huang et al. (2015) determined the in vivo pharmacokinetic profile of bufadienolides (resinobufagin, bufalin, gamabufotalin, aerobufagin, and bufotalin) from Shexiang Baoxin pill following oral administration. These compounds were rapidly absorbed and reached high plasma concentrations, except for bufotalin whose tissue with higher concentrations was the intestine (Huang et al. 2015).

On the other side, compounds with such permeability may penetrate the blood–brain barrier (BBB), a physiological obstacle constituted by a capillary basement membrane and cellular elements (endothelial cells, pericytes, and astrocyte end-feet) responsible for maintaining homeostasis for neuronal functions and providing neuronal tissue with nutrients, and defending the CNS against toxic insults and pathogens (Dotiwala et al. 2023). So, crossing the BBB is a minimum requirement for a compound with CNS activity (Wu et al. 2023; Konig et al. 2025), but a dangerous property for toxins. So, neuroscientists consider it should exhibit specific physicochemical characteristics, including (i) molecular weight < 400 Da; (ii) Log P value close to 2.0; (iii) < 10 hydrogen bonds; and (iv) the compound should be in a non-ionic form or naturally basic (Rankovic 2015; Hamed et al. 2023). These features partially explain the intermediate ability of MBG to cross the BBB, especially because its molecular weight is above the acceptable limit. Within in silico conditions, it would be pharmacologically inactive in the CNS (C_brain_/C_plasma_ < 0.40). However, amphipathic vehicles as DMSO (used here) and polyethylene glycol, commonly applied in preclinical research as co-solvents, enhance the BBB permeability and accelerate drug penetration into the CNS (Iwen and Miller 1986; Ziylan et al. 1988). Kumari et al. (2018) demonstrated that animals receiving pentylenetetrazol in DMSO 0.1% exhibited more seizures and histopathological damage if compared to those treated with pentylenetetrazol without DMSO.

This study provides the first characterization of MBG neurotoxic effects. Prior in vivo pharmacokinetic analyses with bufotalin revealed significant brain concentrations 30 min after caudal intravenous injection (Yu and Hou 2011). Animal models of preeclampsia demonstrated that MBG can impair microvascular endothelial permeability (specifically in the lungs) through the activation of apoptotic pathways and disruption of cellular junctions (Uddin et al. 2009), which could facilitate its body distribution. Consequently, it is believed that MBG may induce alterations in the BBB, a hypothesis later confirmed byin vitro studies using human cerebral endothelial cells (Uddin et al. 2012; Ing et al. 2014; Pantho et al. 2024).

Considering the antiproliferative potential of MBG, including its activity against refractory tumor cells to anticancer agents (Machado et al. 2018; Ferreira et al. 2023), its effect on P-glycoprotein (P-gp) was also evaluated, but no activity was observed. Other bufadienolides, as bufalin, blocked this transporter in cerebral endothelial cells (Mahringer et al. 2010) and cinobufagin inhibits P-gp activity through a non-competitive mechanism (Yuan et al. 2017), which suggests that each bufadienolide presents P-gp different inhibitory capacity.

Marinobufagin was also identified as an inhibitory substrate of CYP3A4 enzyme, a monooxygenase isoform that metabolizes a wide range of drugs. It is well established that there is a correlation between the high lipophilicity of a substrate and its binding affinity to the enzyme (Zanger and Schwab 2013; Kiani and Jabeen 2019), which partially explains its preference for MBG. In vitroand in vivo studies have demonstrated that bufalin (Li et al. 2009a) and cinobufagin (Ma et al. 2011) can inhibit microsomal CYP3A4 activity, and Li et al. (2009b) and Weng et al. (2011) report that bufadienolides possess structural features that facilitate their hydrolysis.

In this way, drug-likeness prediction is a tool used to evaluate the molecular and physicochemical similarity of a compound to well-known drugs. To be considered drug-like, candidate molecules should fulfill specific edges defined by different predictive rules. Among them, the “rule of five” (Lipinski’s rule) is widely recognized for establishing key structural parameters of oral bioavailability theoretical prediction. So, compounds have poor absorption/permeability when they do not meet two or more of the following criteria: molecular weight > 500, hydrogen bond donors > 5 (sum of OH and NH groups), hydrogen bond acceptors > 10 (sum of O and N atoms), and log P > 5 (Feher and Schmidt 2003; Lipinski et al. 1997). Meanwhile, MDDR-like rule includes the number of rings, rigid bonds, and rotatable bonds. MBG does not meet the criterion for the number of rotatable bonds, classified as an intermediate structure between drug-like and non-drug-like compound (Oprea 2000). This data indicates a lower flexibility of the molecule, which may increase pharmacotoxicological selectivity for a specific target (Caron et al. 2020).

Conversely, WDI-like rule indicates MBG does not exhibit drug-like properties, since its molecular aspects fall within the upper cut-off limits of 90% and display four violations, and Lead-like rule (Teague et al. 1999) disqualified MBG based on a single violation: high molecular weight. Despite most lead-like molecules are typically smaller, which makes structural optimizations easier for improving potency (Vistoli et al. 2008), this deviation does not primarily reflect limited lead-like properties, but high molecular weight has been associated with unfavorable pharmacokinetic behavior and toxicological liability (Pachla et al. 2023). Anyway, such effects rely on the molecular class, structural features, and the overall physicochemical profile.

The radar of bioavailability indicated favorable overall oral bioavailability attributes of MBG (lipophilicity, solubility, polarity, molecular weight, molecular flexibility, and saturation) (Daina et al. 2017; Stefaniu and Pirvu 2022) and BOILED-Egg model predicts intestinal absorption and BBB penetration and MBG as a likely substrate for P-glycoprotein (Daina et al. 2017; Rajaselvi et al. 2023). Nonetheless, kalanchosides, a subclass of bufadienolides with cytotoxic activity against tumor cells isolated from *Kalanchoe gracilis* (Crassulaceae), were not identified as P-gp substrates (Wu et al. 2006), but they clearly have different cellular targets.

The discrepancy between the BOILED-Egg prediction and the observed central effects of MBG may reflect intrinsic limitations of in silico BBB permeability models. The predictive performance of such models is inherently constrained by the chemical space represented in their training datasets, meaning they can reliably estimate permeability only for compounds structurally and physicochemically like those used in their development (OECD 2014). Given the unique steroidal structure and high lipophilicity of bufadienolides, extrapolation beyond the model’s training domain may reduce predictive accuracy (Delgado-González et al. 2025).

Furthermore, the BBB is a highly dynamic and complex biological interface influenced by active transport mechanisms, efflux proteins, and interindividual physiological variability that are not fully captured by simplified descriptor-based models. Quantitative structure–activity and structure–property relationship (QSAR/QSPR) approaches also depend on the quality and representativeness of experimental data used for model development (Mao et al. 2021; Souza et al. 2024; Alves et al. 2025). Then, many computational tools rely primarily on passive diffusion parameters and do not account for formulation-dependent effects or co-solvent-mediated changes in permeability. Therefore, while the BOILED-Egg model provides useful preliminary insight into passive BBB permeability, discrepancies between computational predictions and in vivo findings, particularly for lipophilic toxins administered with permeability-enhancing vehicles, should be interpreted with caution (Ekins et al. 2024). Similar inconsistencies have been described in studies comparing in silico predictions of BBB permeability for pesticides with experimental human pharmacokinetic data. Then, although some computational models predicted quasi-absence BBB penetration, these results may not correlate with clinical findings in humans (Chedik et al. 2017).

Herein, it was found that MBG interacts with protein kinases. These serine-threonine enzymes play critical roles in cellular development, proliferation, differentiation, and growth, and their dysregulation or overexpression is directly associated with carcinogenesis, tumor progression (Ferreira and Pessoa 2017; Bunardi et al. 2025), and epileptogenesis, since hyperactivation of mTORC1-, mTORC2-, and Akt-mediated signaling pathways, and PTEN loss function in the cortex and hippocampus of epileptic mice contribute to the onset of seizures (Zeng et al. 2009; Cullen et al. 2024).

Moreover, previous studies have demonstrated the inhibitory capacity of bufadienolides against Na^+^/K^+^-ATPase (Bressman et al. 2017; Sousa et al. 2017; Garcia et al. 2019), α-glucosidase, and acetylcholinesterase (AChE) (Xiao et al. 2018), as well as the modulatory activity of a *K. daigremontiana* fraction on plasmin and thrombin (Kolodziejczyk-Czepas et al. 2017, 2018). The AChE inhibitory activity of MBG should enhance cholinergic signaling by prolonging the availability of acetylcholine in the synaptic cleft and random binding to the receptors. Fractions from *R. icterica* and *R. schneideri* parotoid secretions at 10 and 60 µg/mL have also stimulated AChE activity in young chicken neuromuscular preparations (Oliveira et al. 2020) and *Nauphoeta cinerea* cockroaches (Leal et al. 2020), respectively.

Prior investigations have linked AChE activity to seizure and epilepsy pathophysiology (Gnatek et al. 2012; Wang et al. 2021). For instance, in the pilocarpine-induced status epilepticus model, a reduction in AChE activity was observed across various brain regions, including the hippocampus, frontal cortex, and striatum (Freitas 2006) evidenced by electroencephalographic alterations following neostigmine (Joosen and Helden 2007). In this same line, hippocratic screening outcomes following an acute dose of MBG 10 mg/kg in mice were indicative of direct action on the CNS, whose signals were irritability, loss of corneal reflex and muscle tone, difficulty breathing, ataxia, and seizures (Ferreira et al. 2023). The present investigation is also dedicated to underlining how MBG causes convulsions.

Once MBG is a well-established Na^+^/K^+^-ATPase inhibitor (Bagrov et al. 1995; Liu et al. 2012) and this pump is associated with neuronal excitability, seizures, and epilepsy (Silva et al. 2011; Sun et al. 2022), the present study also investigated the modulation of other target channels and neurotransmission pathways. First of all, it was found that the time for seizure onset in MBG-pretreated animals and mortality rate were similar to those receiving morphine, which indicates the disinhibition of hippocampal pyramidal cells (via GABAergic pathway inactivation) induced by opioids has no synergistic effects with MBG (Gholami et al. 2014).

Acetylcholine concentration increase within the synaptic cleft is associated with intense seizure activity, either through hyperactivation of muscarinic M_1_ and M_2_ receptors or AChE inhibition (Nguyen et al. 2024). However, the maintenance of seizure episodes (71%) after atropine treatment suggests that muscarinic activation is not the key mechanism of MBG-induced seizures, given that cholinergic epilepsy-inducing compounds, such as pilocarpine, are inhibited by atropine (Freitas et al. 2006). On the other hand, fewer deaths in the groups treated with morphine and atropine, as well as the surviving animals in other groups with shorter seizure latency periods, clearly propose less neurological and behavioral sequelae induced by MBG (Chen et al. 2023). 

Fluoxetine, clinically used as an antidepressant, has anticonvulsive activity attributed to the increased extracellular concentration of 5-hydroxytryptamine (5-HT) in the synaptic cleft, and activation of 5-HT_3_ and 5-HT_1_ receptors, reducing the intensity and frequency of both focal and generalized seizures, and mitigating oxidative stress and neuronal damage neuroprotection (Pottoo et al. 2019; Sousa et al. 2026). Therefore, the high incidence of seizures in the fluoxetine-treated group (6 out of 7 animals) strongly suggests that this pathway is not involved in the MBG-induced ictogenesis, a similar outcome observed for gabapentin, a VGCC blocker, which did not reduce seizures. As a GABA analogue, gabapentin is traditionally used as an anticonvulsive for simple or complex seizures and neuropathic pain conditions (postherpetic neuralgia, diabetic neuropathy, and chemotherapy-induced algesia) (Wiffen et al. 2017; Benarroch 2021). Since gabapentinoids’ efficacy in these disorders is primarily attributed to the α2δ subunit inhibition of presynaptic VGCCs (reducing neurotransmitter release) (Benarroch 2021), it is believed MBG does not act by these channels.

The involvement of dopaminergic pathways in epileptogenesis is also well established. The activation of D1 (pro-convulsive) and D2 (anticonvulsive) receptors elicit dissimilar responses. Then, D2 antagonistic antipsychotics (e.g., haloperidol) reduce seizure thresholds (Freitas et al. 2006). Although haloperidol chronically suppresses depolarization in the striatum and nucleus accumbens (Lane and Blaha 1987), the effect observed in this study was paradoxical, as it increased seizure latency. On the other hand, despite its low affinity, it also blocks 5-HT_2_ and α_1_ receptors, and its anti-5-HT_2_ excitatory activity may inhibit the propagation and maintenance of seizure episodes (Sourbron and Lagae 2022). Additionally, the interaction of MBG with dopaminergic D2 receptors reveals a dual binding mode, involving both classical orthosteric and site lateral hydrophobic sites. A hydrogen bond with Asp114, a highly conserved residue among aminergic G protein-coupled receptors (GPCRs), represents a vital factor of affinity (Harris et al. 2017). So, the formation of a “hydrophobic clamp” involving Trp100, Ile184, and Leu94 confers substantial stability to the complex. This structural arrangement is particularly relevant because Trp100 is a key regulator of binding kinetics in aminergic receptors and is conserved across other receptor subtypes, such as 5-HT_2A_ and α_1A_-adrenergic receptors (Harris et al. 2017).

Among the groups that received GABAergic neurotransmission-modulating drugs (diazepam and clonazepam), diazepam was more effective to reduce seizures. As seen by molecular docking, the binding of MBG to four distinct sites involving the residues Ser269, Met235, and Ala290 in the interfaces of GABA_A_ receptor subunits may represent a key determinant for receptor modulation (Laverty et al. 2019). Benzodiazepines bind to specific sites on the GABA_A_ receptor, increase its affinity for the neurotransmitter GABA, and enhance CNS inhibitory synaptic responses by the activation of Cl^−^ channels (Miller and Aricescu 2014; Edwards and Preuss 2025). Thus, as positive allosteric modulators, benzodiazepines improve GABA’s inhibitory activity and the brain’s output of excitatory neurotransmitters (including norepinephrine, serotonin, dopamine, and acetylcholine) is reduced (Araújo et al. 2017). Therefore, they are suitable to treat epileptic seizures, ongoing convulsive episodes, and *status epilepticus*. Diazepam, for example, is a first-line drug that prevents permanent neurological damage and physical trauma (Alshehri et al. 2017).

Ketamine- and carbamazepine-pretreated animals neither exhibited seizures nor deaths after MBG injection. Primarily used as anesthetic for both humans and animals, ketamine is a phencyclidine hydrochloride neuroprotective derivative with potent non-competitive antagonist action in the phencyclidine binding site without opposing glutamate in the N-methyl-D-aspartate receptor (NMDA) receptor (Lodge and Mercier 2015), the most extensively studied receptor in the glutamatergic pathway because of its significant etiological role in neuronal excitotoxicity, ictogenesis, and epilepsy (Lason et al. 2013; Rahimi-Madiseh et al. 2022). When the excitatory amino acid NMDA is administered intraperitoneally, animals exhibit hyperactivity and agitation, followed by spasms and generalized tonic–clonic seizures (Stafstrom and Sasaki-Adams 2003), parallel findings to the present study whose animals received MBG, and explains, at least in part, a complete blockade of seizures and deaths by ketamine. Actually, it exhibits anticonvulsive activity in many experimental models and has been investigated as a pharmacological alternative for seizure management (Synowiec et al. 2013; Gibuła-Tarłowska et al. 2025). Actually, molecular docking investigations demonstrated pharmacologically relevant affinity of MBG for the GluN2A and GluN2B subunits. The π–anion interaction with Asp138 in GluN2B, which supports the sensitivity of this site to pH fluctuations (Regan et al. 2019), could theoretically stabilize an open conformation of NMDA channels, particularly under conditions of neuronal depolarization and activation of voltage-gated ion channels.

Voltage-gated sodium channels [VGSCs (Na_v_)] are also implicated in ictogenesis, propagation, and frequency of seizure episodes (Wang et al. 2017). Therefore, pretreatment with carbamazepine, as observed with ketamine, completely prevented the occurrence of seizures (Kobayashi et al. 2020), suggesting the involvement of Na_v_ channels in MBG-induced ictogenesis (beyond the role of glutamatergic NMDA neurotransmission). Moreover, the multimodal interaction of MBG with the Nav1.2 channels observed in docking simulations involved simultaneous sites, selective cavities, and intra- and extracellular access pathways, which insinuates a complex mechanism of channel modulation.

Previously, studies based on behavioral and electrographic investigations displayed similar toxicity following systemic administration of bufadienolide(s). Bufadienolide-loaded nanostructured lipid carriers or a solution of bufadienolides (bufalin, cinobufagin, and resibufogenin) injected parentally (1.32–4.05 mg/kg) caused severe tachypnea, clonic spasms, arrhythmia, and paralysis in all groups, with deaths within a 4-h observation period and LD_50_ values between 2.12 and 3.28 mg/kg. Subsequently, surviving animals restored normal macrophysiological conditions (Li et al. 2010), whose effects can be attributed to the neurotoxicity from accumulation and bufadienolide slower metabolism in the brain.

Bufalin, cinobufagin, and resibufogenin incorporated into lipid microspheres or i.v. injected using an aqueous solution also caused convulsions, tremors, and grooming-like behavior only in rats that received a bufadienolide solution (Weng et al. 2011). It is likely that lipid microsphere formulation has prevented adverse effects by hampering bufadienolides’ ability to reach the CNS. Next, Li et al. (2015) found oral LD_50_ values ranging from 19.6 to 29.4 mg/kg for microemulsions with the same bufadienolides and most animals sharing toxic symptoms. Recently, our group carried out toxicity studies after a MBG single dose, with special attention for the initial 240 min (Ferreira et al. 2023). Such results showed well-defined signals of central activity (ataxia, convulsions, equilibrium loss, mainly) and deaths from 10 mg/kg onwards. All these insights raised concerns about doses due to the narrow therapeutic window (DL_50_ values between 1 and 50 mg/kg) and corroborate MBG-induced ictogenesis directly.

As indicated here by in vivo studies using VGCCs’ and VGSCs’ blockers (carbamazepine and gabapentin, respectively), it is very likely that activation of Ca^2+^ and Na_v_ channels by MBG may lead to neuronal injury induced by depolarization, creating an ideal environment for the onset of seizures. Considering that Na_v_1.2 is predominantly expressed in excitatory neurons in neocortex and hippocampus (Spratt et al. 2021), in silico results also confirmed MBG molecular interactions with Na_v_1.2 channels mediated by different ways (van der Waals, hydrophobic residues, hydrogen bonds, and electrostatic interactions). Moreover, electrophysiological studies on hippocampal neurons from resibufogenin-treated neonatal rats revealed that it (i) increased the amplitude peak of I_Na⁺_ (Na^+^ inward current), suggesting that the potential binding site in Na_v_ channels may be situated near the extracellular end of the S4 transmembrane segment in the α subunit where the channel’s voltage sensor resides, confirming the likelihood of MBG bind to the extracellular site of Na_v_1.2; (ii) decreased the recovery time from I_Na⁺_ inactivation, increasing neuronal excitability and reducing refractoriness; and (iii) rises the Ca^2+^ intracellular concentration ([Ca^2+^]ᵢ) probably as a result from the increase in I_Na⁺_ and the reverse role of physiological Na^+^-Ca^2+^ exchanger (Hao et al. 2011). Anyway, a distinctive feature of bufadienolides accounts for toxicodynamic diversity, whose bioactivity depends on the specific receptors they interact with (Carullo et al. 2023).

Considering the inhibition of the Na^+^/K^+^-ATPase disrupts ionic homeostasis, leading to intracellular Na^+^ accumulation and membrane depolarization, whose higher intracellular Na^+^ may also reverse the Na^+^/Ca^2+^ exchanger, promoting intracellular Ca^2+^ accumulation and secondary excitotoxicity, the collapse of the Na^+^ gradient also compromises neurotransmitter reuptake mechanisms, particularly for glutamate clearance, which enhances excitatory synaptic transmission (Gadsby et al. 2012; Arnaiz et al. 2014). In this context, the computationally predicted interactions of MBG with Na_v_ channels and NMDA receptors may represent complementary or amplifying mechanisms that further potentiate excitatory neurotransmission. Therefore, Na^+^/K^+^-ATPase inhibition and modulation of excitatory Na_v_ channels (Arnaiz et al. 2014) should not be viewed as mutually exclusive, but rather as potentially convergent processes contributing to MBG-induced seizures. Supporting this integrative mechanism, previous studies have also demonstrated that endogenous cardiotonic steroids such as endobain exert neurotoxic effects through Na^+^/K^+^-ATPase inhibition, leading to NMDA receptor activation (Reines et al. 2001; Reines et al. 2004; Pivovarov et al. 2018).

Other molecular targets could have been included in both in silico and in vivo analyses. Nevertheless, we focused on receptors more directly associated with clinical background of seizures. In a molecular point of view, in silico resolutions of 3.0 Å (or poorer) make side-chain density less distinct and the resolution limit is noted when it is pondered the accurate positioning of specific side chains or ligands. Unfortunately, higher-resolution structures were not available for 6J8E structure, for example.

As main limitation of this study and considering the high lipophilicity (Log P: 3.13) and poor water solubility (Log S: − 3.99) of MBG, it was not possible to assess its neurotoxicity without a co-solvent (DMSO). Herein, it is important to emphasize that the central activity of bufadienolides is dose-dependent under identical conditions using DMSO 5% (Ferreira et al. 2023), leading to behavioral alterations and reinforcing MBG central pharmacotoxicological effects. Although behavioral assessments were not conducted beyond the 4-h observation period described in the study, we have considered this time based on previous studies showing that bufadienolides’ actions occur acutely in the early hours (Li et al. 2010, 2015; Ferreira et al. 2023). Furthermore, efforts minimized the use of animals in accordance with the principles of the 4Rs (Ferreira et al. 2025), reason by which they were chosen key pharmacological targets.

## Conclusions

Molecular docking studies emphasized the interaction of MBG with ictogenesis-related targets, confirmed by reducing in vivo seizures and death of animals pretreated with pharmacological blockers for D2 and NMDA receptors and Na_v_1.2 channels, mainly. It was shown that affinity of MBG for excitatory targets is essential for neuronal excitability and onset of seizures and that in silico-predicted interaction with GABA_A_ receptors is probably involved in bufadienolide-induced lethality. The findings also suggest a potential interaction of MBG with NMDA receptors under physiological conditions. In silico and in vivo outcomes highlights BBB-crossing capacity as a dangerous property of (neuro)toxins, and violations of drug-likeness rules can be correlated with MBG-associated venomousness, which allows an indirect association with toxicological risk, but it also draws attention to biotechnological purposes. So, the present study characterized MBG-induced seizures using non-clinical pharmacological tools, introducing a novel perspective on the toxicological profile of bufadienolides, and proposing MBG as a promising chemical option to understand ictogenesis and mechanism(s) of new anticonvulsive agents, since timely diagnosis and adequate treatment can ensure that most affected people have reduced episodes.

## Supplementary Information

Below is the link to the electronic supplementary material.

ESM 1 (DOCX 243 KB)

## Data Availability

The data that support the findings of this study are available from the corresponding author (P.M.P. Ferreira) upon reasonable request.
