# Supplementary material for: Central toxic effects of an antitumoral bufadienolide: In silico targets and in vivo findings
Source: Naunyn Schmiedebergs Arch Pharmacol. 2026 May 26;399(11):17693–713. doi: 10.1007/s00210-026-05462-y (PMC13503435; doi:10.1007/s00210-026-05462-y)
Supplement: Supplementary file 1 [file 210_2026_5462_MOESM1_ESM.docx]

Root mean square deviation (RMSD) of the ligand from the redocking analysis.

| Receptor | RMSD (Å) | Structure |
| --- | --- | --- |
| Na_v_1.2 | 1.096 |  |
| D2 | 0.181 |  |
| GABA_A_ | 0.953 |  |
| NMDA Grin2A | 1.212 |  |
| NMDA Grin2B | 1.225 |  |
